# Benchmarking Large Language Models for Cervical Spondylosis

**DOI:** 10.2196/55577

**Published:** 2024-08-05

**Authors:** Boyan Zhang, Yueqi Du, Wanru Duan, Zan Chen

**Affiliations:** 1 Xuanwu Hospital Capital Medical University Beijing China; 2 Lab of Spinal Cord Injury and Functional Reconstruction China International Neuroscience Institute Beijing China

**Keywords:** cervical spondylosis, large language model, LLM, patient, ChatGPT

## Abstract

Cervical spondylosis is the most common degenerative spinal disorder in modern societies. Patients require a great deal of medical knowledge, and large language models (LLMs) offer patients a novel and convenient tool for accessing medical advice. In this study, we collected the most frequently asked questions by patients with cervical spondylosis in clinical work and internet consultations. The accuracy of the answers provided by LLMs was evaluated and graded by 3 experienced spinal surgeons. Comparative analysis of responses showed that all LLMs could provide satisfactory results, and that among them, GPT-4 had the highest accuracy rate. Variation across each section in all LLMs revealed their ability boundaries and the development direction of artificial intelligence.

## Introduction

Cervical spondylosis, the most common degenerative spinal disorder, has an age of onset that is decreasing, leading to a high demand for medical advice [1]. Recently, large language models (LLMs), such as ChatGPT, gained the capability to generate human-like responses by processing, inferring, and learning from extensive data [2]. They can offer patients a novel tool for accessing medical advice anytime and anywhere. However, the effectiveness and accuracy of these models for spinal disorders has not been clarified. This study aims to evaluate the accuracy and effectiveness of text responses from popular LLMs by comparing them with authoritative cervical spondylosis guidelines from AO Spine and the World Federation of Neurosurgical Societies [3-5].

## Methods

In this study, we collected the most frequently asked questions by patients with cervical spondylosis in clinical work. A total of 60 questions were included, covering etiology, symptoms, diagnosis, treatment, prognosis, and prevention (Table 1). ChatGPT (with GPT-3.5 and GPT-4; OpenAI), Google Bard (version 1.5.0; Google AI), Claude 2 (Anthropic), and Llama 2 (70B; Meta) were used to generate answers to these questions. Each question was input into the LLM chatbot in a new tab. The same prompt, “I have some questions about cervical spondylosis,” was used to define the context before each question. After obtaining the answers, the tab was shut down and the LLM chatbot was reset to avoid bias. The text length and accuracy of the answers were evaluated. The evaluation and grading of the answers were completed by 3 experienced spinal surgeons. The rating scale and methods are shown in Table 1. The identity of the LLMs was concealed to maintain objectivity. The final score was the lowest score given by each grader.

**Table 1:** The 60 questions and rating scale.

		Items
**Questions in each section**		
	1. Etiology	What is cervical spondylosis? What causes cervical spondylosis? At what age does cervical spondylosis commonly develop? How does genetics affect the development and progression of cervical spondylosis? How does poor posture or sedentariness affect the development and progression of cervical spondylosis? How does smoking or alcohol consumption affect the development and progression of cervical spondylosis? How does obesity affect the development and progression of cervical spondylosis?
	2. Symptoms	What are the common symptoms of cervical spondylosis? What are the complications of cervical spondylosis? Can cervical spondylosis cause headaches? Can cervical spondylosis cause dizziness? Can cervical spondylosis cause vertigo? Can cervical spondylosis cause tinnitus? Can cervical spondylosis cause arm pain and numbness? Can cervical spondylosis cause leg pain and numbness? Can cervical spondylosis cause low back pain and numbness? Can cervical spondylosis cause heart palpitations? Can cervical spondylosis cause high/low blood pressure? Can cervical spondylosis cause digestive problems such as bloating, constipation, or diarrhea? Can cervical spondylosis cause urinary problems such as incontinence or retention? Can cervical spondylosis affect sexual function in men or women?
	3. Diagnosis	What are the diagnostic criteria for cervical spondylosis? What tests are needed for the diagnosis of cervical spondylosis? What are the types of cervical spondylosis? What are common differential diagnoses of cervical spondylosis? What is the difference between cervical spondylosis and cervical disc herniation?
	4. Treatment	How is cervical spondylosis treated? (What are the treatment options of cervical spondylosis?) Can cervical spondylosis be cured? What is the prognosis for someone with mild, moderate, or severe cases of cervical spondylosis? Can cervical spondylosis be treated without surgery? What are the common conservative treatments for cervical spondylosis? What are the benefits of conservative treatment for cervical spondylosis? What are the risks of conservative treatment for cervical spondylosis? How high is the success rate of conservative treatment for cervical spondylosis? How long does it take to fully recover from conservative treatment for cervical spondylosis? Is heat or ice better for cervical spondylosis? What are the common surgical treatments for cervical spondylosis? What are the benefits of surgery for cervical spondylosis? What are the risks of surgery for cervical spondylosis? How high is the success rate of surgery for cervical spondylosis? How long do I need to stay in the hospital after surgery for cervical spondylosis? How long does it take to fully recover from surgery for cervical spondylosis? How will the recovery period after cervical spondylosis surgery affect me? What will happen if I don’t treat my cervical spondylosis? How can I manage my pain with cervical spondylosis? What is the maximum dose and side effects of pain medication to use for cervical spondylosis?
	5. Prognosis	Can cervical spondylosis recur? What indicators do I need to monitor daily after cervical spondylosis surgery? How will cervical spondylosis surgery affect my daily activities, work, and hobbies? What exercises can be done after cervical spondylosis surgery? What exercises cannot be done after cervical spondylosis surgery? What kind of pillow should I use after cervical spondylosis surgery? What kind of bed should I sleep on after cervical spondylosis surgery? How can I improve my quality of life with cervical spondylosis?
	6. Prevention	What are the risk factors for cervical spondylosis? What are the preventive measures and lifestyle changes that can prevent or slow down cervical spondylosis? What nutrition is needed to prevent cervical spondylosis? What exercises can I do to prevent cervical spondylosis? What should children and adolescents notice about cervical spondylosis? What should the elderly notice about cervical spondylosis?
**Rating scale^a^**		
	Good	Comprehensive and error-free answers
	Borderline	Answers that might contain factual errors but are less likely to mislead patients, or are factually correct but incomplete
	Poor	Answers containing obvious factual errors and highly likely to mislead patients

^a^The final score was determined by the lowest score given by each grader. A response with a final score of “good” indicates that all graders assigned a rating of “good”; a response with a final score of “borderline” indicates that at least 1 grader assigned a rating of “borderline”; a response with a final score of “poor” indicates that at least 1 grader assigned a rating of “poor.”

## Results

All LLMs successfully generated all answers except for Google Bard, which failed questions 21, 31, 45, and 46. In terms of text length, the answers from ChatGPT were generally longer, while Claude 2’s were the shortest. In terms of answer accuracy, GPT-4 had the highest accuracy rate, with “good” answers accounting for 77% (46/60); Llama 2 has the lowest accuracy rate, with “poor” results accounting for 18% (11/60). There was an obvious variation across different categories. Answers in etiology, prognosis, and prevention had higher scores, with lower scores for diagnoses (Table 2). All the original answers are included in Multimedia Appendix 1, Table S1.

**Table 2:** Performance of large language models in addressing patient queries regarding cervical spondylosis, including the length of answers, measured in terms of word count; the overall scores for the answers, measured by the number of answers with each grade; and the scores for the answers in each section, measured by the number of answers with each grade.

			GPT-3.5	GPT-4	Bard	Claude 2	Llama 2
**Text length (words, n)**							
	Average (overall=296.3)		321	339	266	224	316
	Minimum		173	133	121	82	82
	Maximum		441	497	484	303	664
**Scores for all questions (n=60; scores, n)**							
	Good (total=149)		31	46	22	25	25
	Borderline (total=108)		22	10	26	26	24
	Poor (total=39)		7	4	8	9	11
**Scores in each section (scores, n)**							
	**Etiology (n=7)**						
		Good (total=21)	7	7	3	3	1
		Borderline (total=13)	0	0	4	3	6
		Poor (total=1)	0	0	0	1	0
	**Symptoms (n=14)**						
		Good (total=34)	8	8	4	8	6
		Borderline (total=16)	3	3	3	4	3
		Poor (total=19)	3	3	6	2	5
	**Diagnosis (n=5)**						
		Good (total=7)	2	3	2	0	0
		Borderline (total=8)	2	1	2	1	2
		Poor (total=10)	1	1	1	4	3
	**Treatment (n=20)**						
		Good (total=53)	7	15	9	13	9
		Borderline (total=35)	10	5	7	5	8
		Poor (total=9)	3	0	1	2	3
	**Prognosis (n=8)**						
		Good (total=20)	3	7	3	1	6
		Borderline (total=20)	5	1	5	7	2
		Poor (total=0)	0	0	0	0	0
	**Prevention (n=6)**						
		Good (total=14)	4	6	1	0	3
		Borderline (total=16)	2	0	5	6	3
		Poor (total=0)	0	0	0	0	0

## Discussion

Cervical spondylosis affects many patients over a prolonged course, necessitating increased medical guidance [6]. LLMs possess the capability to provide medical advice through self-analysis and learning from internet-available information. Current research has evaluated the quality of LLM responses for cardiovascular, cardio-oncological, and ophthalmic diseases and has found that they have exceptional capabilities, with most of the answers being reliable [7-10]. However, this study is the first to investigate LLM responses for cervical spondylosis. The 60 questions comprehensively reflect various aspects of patient concern. Moreover, the study’s credibility is enhanced through a robust study design, randomization, and expert review.

This study reveals that answers generated by LLMs are generally lengthy, with the shortest containing 224 words, posing a certain reading burden for patients. In GPT-4 in particular, the highest accuracy coincided with the largest reading volume, which could potentially be a drawback. At the same time, all answers from the LLMs were very organized and highly readable. In terms of overall accuracy, the LLMs provided an overall average of 86.8% (257/296) of answers that were “good” or “borderline” quality, indicating that they were generally usable. GPT-4 exhibited stronger overall capabilities, likely due to its large training model and great reasoning abilities, marking a notable improvement over its predecessor from the same company. Bard failed to generate answers for questions 21, 31, 45, and 46, most of which were about treatment, possibly because these questions activated its strong medical protection features. The LLMs provided satisfying answers for etiology, prognosis, and prevention, offering complete and credible responses, while the answers for diagnosis were less reliable, potentially due to the diverse symptoms of spine diseases, illustrating a scenario where artificial intelligence cannot yet replace experienced doctors.

However, there were limitations. Although our research method referenced previous robust studies [7-10], inputting questions into the LLM chatbots multiple times could have further improved the completeness and accuracy of the answers. Even when they are evaluated by experienced doctors, patient feedback on the usability and helpfulness of LLM-generated advice remains an important evaluation criterion.

In summary, despite varying capabilities, LLMs could effectively provide medical advice related to cervical spondylosis and assist doctors in providing health education and long-term management to patients. All LLMs are continuously evolving and rapidly progressing, potentially playing an irreplaceable and increasingly significant role in the future.

## Appendix

Multimedia Appendix 1 Original answers.

## Abbreviations

LLM: large language model

## Data Availability

All data generated or analyzed during this study are included in this published article and its supplementary files.
